# Microstructure and Grain Orientation Evolution in SnPb/SnAgCu Interconnects Under Electrical Current Stressing at Cryogenic Temperature

**DOI:** 10.3390/ma12101593

**Published:** 2019-05-15

**Authors:** Xing Fu, Yunfei En, Bin Zhou, Si Chen, Yun Huang, Xiaoqi He, Hongtao Chen, Ruohe Yao

**Affiliations:** 1 School of Electronics and Information, South China University of Technology, Guangzhou 510640, China; fuxing@ceprei.com; 2 China Science and Technology on Reliability Physics and Application of Electronic Component Laboratory, Guangzhou 510610, China; enyf@ceprei.com (Y.E.); zhoubin@ceprei.com (B.Z.); chensi@ceprei.com (S.C.); huangyun@ceprei.com (Y.H.); hxq@ceprei.com (X.H.); 3 China Electronic Products Reliability and Environmental Testing Institute, Guangzhou 510610, China; 4 Shenzhen Graduate School, Harbin Institute of Technology, Shenzhen 518055, China; chenht@hit.edu.cn

**Keywords:** cryogenic, electromigration, grain orientation, IMC, EBSD

## Abstract

Electromigration was characterized at the cathode Cu/solder interface—without the effect of Joule heating—by employing scanning electron microscopy (SEM) and electron backscatter diffraction (EBSD) analyses. Rapid (Cu_x_,Ni_1−x_)_6_Sn_5_ intermetallic compound (IMC) growth was observed at the anomalous region at the cathode end due to the effect of current crowding. The abnormal isotropic diffusion and parallel distribution of Pb were characterized in an ultra-low temperature environment in a monocrystalline structure stressed at −196 °C. The interesting results were attributed to crystallographic transformation due to the simultaneous effect of cryogenic and electrical stressing. The diffusion behavior of Pb atoms in face-centered cubic lattices performed isomorphism. As a result, Pb atoms of the bump gathered at the high-energy grain boundaries by diffusing through the face-centered cubic lattices around the long grain boundary, eventually forming a long-range distribution and accumulation of Pb elements. Our study may provide understanding of cryogenic electromigration evolution of the Cu/solder interface and provide visual data for abnormal lattice transformation at the current stressing.

## 1. Introduction

Deep-space exploration necessitates an astrovehicle of great reliability over an extensive range of temperatures, for instance, under the conditions of Moon (77–423 K), Mars (133–293 K) and Giant Planets (133–653 K) [1,2]. Solder interconnects, applied in microelectronics, can prove problematic at cryogenic temperatures (CT) [3]. Solder alloys are used as structural materials, and the joints electrically and mechanically connect electronic components to printed circuit boards (PCBs) [4,5]. The performance and quality of the interconnects are critical to the overall functionality of the electronic components [6,7,8,9]. Sn-based microbumps undergo a transition from ductile to brittle at CT, resulting in brittle cracks that can lead to catastrophic failure of electronic components. Although industrial production processes are beginning to move away from Pb–Sn solder, the high-reliability areas of aerospace still use lead-tin solder. Due to increasing current densities required for high-performance, multi-functional, and miniaturized electronic devices, electromigration (EM) is becoming a crucial issue for the reliability of solder interconnects [10,11,12]. When the current density is greater than 1.0 × 10^4^ A/cm^2^, the intermetallic compound (IMC) is driven by the electron wind to migrate from the cathode to the anode, resulting in a polar effect of IMC growth [13]. Void propagation at the cathode end and extrusion at the anode end occur frequently until a final EM failure eventuates [14,15,16]. 

EM is affected by the crystallographic structure of Sn-based solder interconnects [17,18]. The most widely used lead-free solders in electronic packaging are Sn-based solder alloys with a β-Sn matrix, such as Sn–Ag and Sn–Ag–Cu [19,20]. Therefore, the characteristics of the interconnects are determined by β-Sn, which exhibits strong anisotropy in multiple aspects, such as mechanical, thermal, electrical, and diffusion properties, due to its body-centered tetragonal crystal structure [21,22,23,24]. The migration and formation of (Cu_x_,Ni_1−x_)_6_Sn_5_ IMCs has been characterized as the main EM failure of solder interconnects, however, the results of IMC growth have been confused with the results of thermomigration (TM). In the actual engineering application process, the tin-based solder micro-interconnect structure changes its internal lattice structure during the process of temperature drop, resulting in a so-called gray tin or tin pest (α-Sn) parasitic phase [25]. Consequently, the mechanical properties and durability of the solder joint are severely impaired. The Sn lattice structure theoretically undergoes a transition from β-Sn to α-Sn at temperatures below 13.2 °C. Some studies have also pointed out that the subcooling temperature required for this transition in practical applications is about a dozen degrees lower than 13.2 °C [25]. In general, the low temperature factor can increase the driving force and the free energy difference ΔG [26]. Conversely, the low temperature factor can reduce the thermal energy of the lattice and the atom migration speed [26]. Therefore, whether or not a lattice transition occurs depends on which force is dominant. The addition of some insoluble metal elements such as Pb, Zn, Al, Mg, and Mn can effectively accelerate the transformation [27,28]. Thus, solder consisting of mixed ingredients also increases the risk of tin pests. In this paper, self-mixed Pb-containing solder joints are used to characterize electromigration inside interconnected solder joints of an α-Sn lattice structure. The difference in IMC migration behavior at low temperatures and at room temperature or under heating was found. Relevant scholars have characterized the degradation of mechanical properties related to the lattice transformation of Sn, but did not conduct detailed research and characterization of the causes of migration of IMCs [29]. In this paper, the characterization of the IMC migration path was carried out, and its migration mechanism was also analyzed. This study can provide meaningful experimental data for the reliability such components in deep-space environments.

In this study, SnAgCu/SnPb microbumps were fabricated and EM tests at CT were conducted under a current density of 2.5 × 10^3^ A/cm^2^. The effect of grain orientation on the electromigration (without the effect of thermomigration) of microbumps was investigated in terms of Pb atoms and IMC migration at CT. Electron backscatter diffraction (EBSD) technology was utilized to investigate the effect of grain orientation and grain boundaries on the migration of Pb atoms and IMCs. 

## 2. Experiment

**Figure 1:**
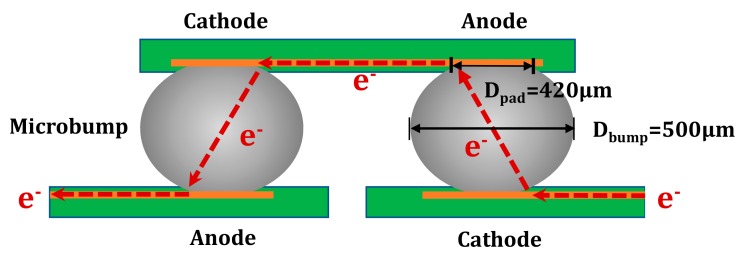
Cross-sectional schematic map of a piece of solder sandwiched between two chips having Au/Ni/Cu/ trilayer films.

A three-plate overlap method was applied to make a simulation chip, as shown in Figure 1. The bump diameter was 500 μm and the Cu pad size was 420 μm. The solder balls used in this paper were self-sintered in the laboratory and based on the content of metal elements required for space environmental testing. The composition of the solder bumps was Sn3.0Ag0.5Cu, SnPb (Pb content is 22.46%) and SnPb (Pb content is 37%). Solder balls of self-matching composition were placed in the center of the pad under the wettability of the flux. The bump was implanted using a flip-chip bonding machine, equipped with a high-precision alignment device, under the protection of nitrogen. The upper and lower sides of the samples were simultaneously heated by hot air to maintain a peak temperature of 260 °C for 60 seconds. The cooling rate was set to 4 °C/s. A self-made micro-interconnect structure of a single flip-chip solder joint was fabricated. The microbumps were stressed under the electrical current density of 2.5 × 10^3^ A/cm^2^ at −196 °C and room temperature at 25 °C. After grinding and polishing, ion milling was performed by applying argon ion source bombardment to remove surface stress layers with the thickness set to approximately 10 μm. The microbumps were analyzed using backscattered electron images, while the Sn grain orientation was examined using EBSD orientation image mapping (OIM) in the normal direction. Image processing was processed with TSL OIM Analysis 7.0 (AMETEK, Inc). The relationship between migration of Pb atoms and Sn grain structure was described in detail later in this study.

## 3. Results and Discussion

**Figure 2:**
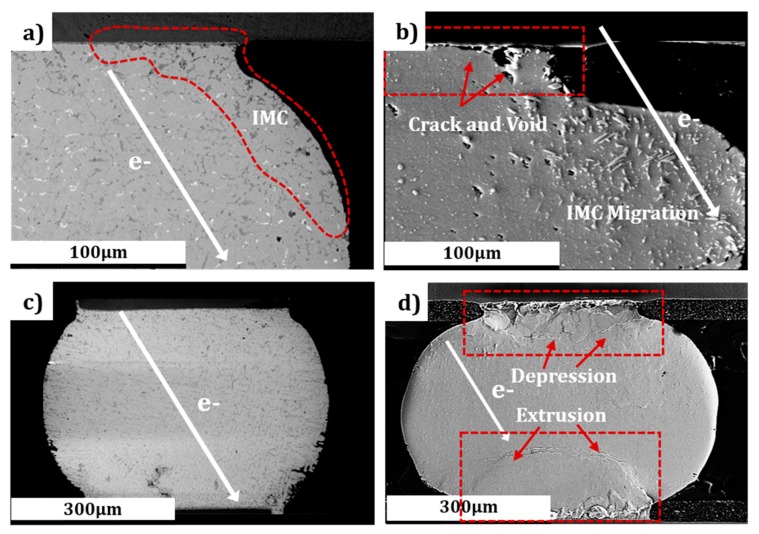
Scanning electron microscopy (SEM) micrographs of microstructures in electromigration (EM) failure at cryogenic temperatures (CT) and room temperature (RT). (**a**) and (**c**) are SEM images of Sn3.0Ag0.5Cu microbumps after stressing at the current density of 2.5 × 10^3^ A/cm^2^ for 304 h at CT (−196 °C); while (**b**) and (**d**) are SEM images of the Sn3.0Ag0.5Cu microbumps after stressing at the current density of 2.5 × 10^3^ A/cm^2^ for 274 h at RT (25 °C).

The comparison tests both proved the occurrence of EM at the cathode interfaces of the two bumps at CT (−196 °C) and room temperature (RT) (25 °C). Figure 2a,c shows the micromorphology of the Sn3.0Ag0.5Cu microbumps after stressing at the electrical current density of 2.5 × 10^3^ A/cm^2^ for 304 h at CT (−196 °C). In contrast, Figure 2b,d depicts the scanning electron microscopy (SEM) images of the Sn3.0Ag0.5Cu microbumps after stressing at the same current density of 2.5 × 10^3^ A/cm^2^ for 274 h at RT (25 °C). Migration of (Cu_x_,Ni_1−x_)_6_Sn_5_ IMCs (identified by Energy Dispersive Spectrometer) from the cathode into the bump under current stressing was observed in both bumps, as shown in Figure 2a,b. IMC migration from current stressing has previously been reported [30], and this finding is reflected in Figure 2. However, the amount of IMC migration in Figure 2b (RT test) was much greater than that of Figure 2a (CT test). As can be seen in Figure 2b, a serrated interface with a layer of voids exists, and the cathode end is seriously decomposed. Contrastively, the cathode interface of the bump stressed at CT remained intact, except for a few voids. The reason for this disparity is attributable to an EM/TM-induced coupling effect at RT, while current stress acts as the dominant driving force for IMC migration in the cryogenic environment. In comparison to Figure 2c, a significant polarization effect may be characterized in Figure 2d due to the effect of EM. The pseudo-color image of local microstructures in Figure 2d shows depression and extrusion resulting from polarization effects. Accordingly, the solder/Cu pad interface at the cathode stressing remained basically intact, and fewer IMCs migratied from the cathode to the anode, as shown in Figure 2c.

**Figure 3:**
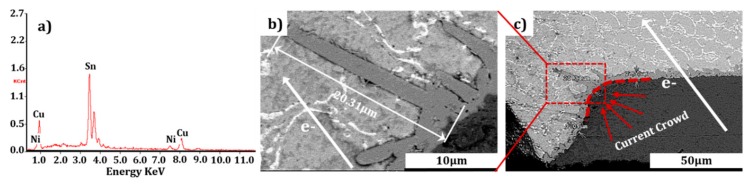
Energy spectrum and SEM images of the rapid growth of (Cu_x_,Ni_1−x_)_6_Sn_5_ IMC at the cathode end in the SnPb (Pb content is 22.46%) microbump after stressing at the current density of 2.5 × 10^3^A/cm^2^ for 100 hr at CT (−196 °C). (**a**) is EDS image; (**b**) is Partial image; (**c**) is Panoramic image.

Although EM did not emerge strikingly at CT, rapid IMC growth was observed at the anomalous region of the cathode interface as a result of the electrical current stressing. Figure 3a is a partially enlarged SEM image showing the IMC morphology detail of Figure 3b. This SnPb (Pb content is 22.46%) bump was stressed at current density of 2.5 × 10^3^ A/cm^2^ for 100 hours. The area with abrupt changes in geometry can be seen at the cathode/Cu interface, where IMC orientation and rapid growth occurred as a result of continuous electrical current stressing. The length of the (Cu_x_,Ni_1−x_)_6_Sn_5_ (identified by EDS) IMC was measured up to 20.31 μm. The anomalous size of the IMC may be attributed to the current crowding effect. The growth of (Cu_x_,Ni_1−x_)_6_Sn_5_ were accelerated rapidly. Such a mechanism has been similarly reported, with current crowding occurring at the entrance of the current (or electron) flow from the conducting trace to the solder bump, accelerating EM damage [30].

The driving force propelling the rapid (Cu_x_,Ni_1−x_)_6_Sn_5_ IMC growth at this region was the rapid migration of Cu atoms, forced by the electron wind. The electrical force acting on the Cu atoms was taken to be main driving force, as illustrated in the equation below proposed by Huntington and Grone [31]:

(1) $$F_{\mathrm{em}}=Z^{*}eE=\left(Z_{\mathrm{el}}^{*}+Z_{\mathrm{wd}}^{*}\right)eE$$

**Figure 4:**
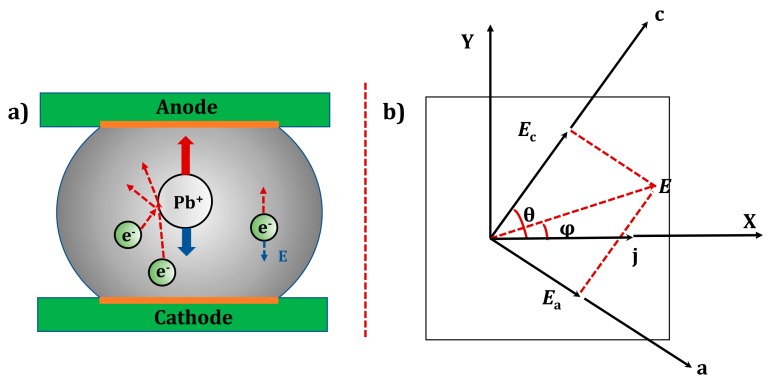
Schematic maps of the dynamics and migration direction in electromigration. (**a**) Diagram of momentum exchange in electromigration; and (**b**) cross section of a α-Sn grain.

Here, e is the charge of the electron, *E* is the electric field (*E* = *ρj*, where *ρ* is the resistivity and *j* is the current density), and $Z^{*}$ is the effective charge number of EM. $Z_{\mathrm{el}}^{*}$ can be interpreted as the nominal valence of the diffusing ions ignoring the shielding effect. $Z_{\mathrm{el}}^{*}eE$ is regarded as the direct force. $Z_{\mathrm{wd}}^{*}$ is a number of charges representing the momentum exchange. $Z_{\mathrm{wd}}^{*}eE$ is called the electron wind force, and it is commonly found to be much bigger than the direct force in EM. Therefore, the Pb/Cu atoms were forced to migrate in the same direction as the electrons, as shown in Figure 4a. Consequently, copper atoms migrated into the bumps and recombined with the Sn atoms in the bumps to form (Cu_x_,Ni_1−x_)_6_Sn_5_ IMC. The microstructure of the migration was characterized by the growth of (Cu_x_,Ni_1−x_)_6_Sn_5_ IMC, as shown in Figure 3a,b. 

In Figure 4b, the cross section of a α-Sn grain is illustrated. It has a face-centered cubic structure and its c-axis is supposed to be making an angle *θ* with the x-axis. The angle between the a-axis and the x-axis is (90° -*θ*), while the b-axis is normal to the plane of the figure. *j* is the stressed current density of the electrons. Note that the resistivity along the a-axis, b-axis and c-axis is the same in the α-Sn lattice. Due to the isotropy of resistivity, the electrical field *E*_a_ = *E*_a_ = *E*_c_ = *ρ*_a_*j*_a_. In this study the tin-based microbump was stressed at CT, and the grain lattice was transferred into the α-Sn lattice with the isotropic properties along all the axes. Consequently, the magnitude of *ρ*_c_*j*_c_ is the same as *ρ*_a_*j*_a_ in α-Sn lattice [32].

(2) $$\cos\varphi =\frac{\rho_{c}\cos^{2}\theta +\rho_{a}\sin^{2}\theta }{\sqrt{{\left(\rho_{a}\sin\theta \right)}^{2}+{\left(\rho_{c}\cos\theta \right)}^{2}}}$$

The above equation represents the magnitude of the angle *φ* between the electrical-field and the stressed electron density. The equation indicates that if *θ* = 0° and *θ* = 90°, then *φ* = 0°, or in these cases *E* will be parallel to *j*. Consequently, the diffusivity of metal atoms in the α-Sn lattice (face-centered cubic structure) performed anisotropic migration along the a, b, and c axes, regardless of the angle φ.

**Figure 5:**
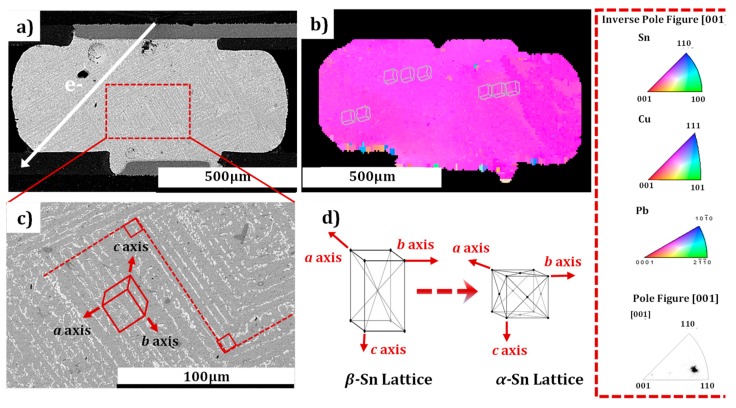
Microstructure images of the microbump with monocrystalline structure stressed at CT. (**a**) and (**c**) are SEM images of the SnPb (Pb content is 22.46%) microbump after stressing at the current density of 2.5 × 10^3^ A/cm^2^ for 100 h at CT (−196 °C); (**b**) corresponding inverse pole figure of the microbump in Figure 3a; and (**d**) schematic diagram of the lattice structure transition from β-Sn (at RT) to α-Sn (at CT).

**Figure 6:**
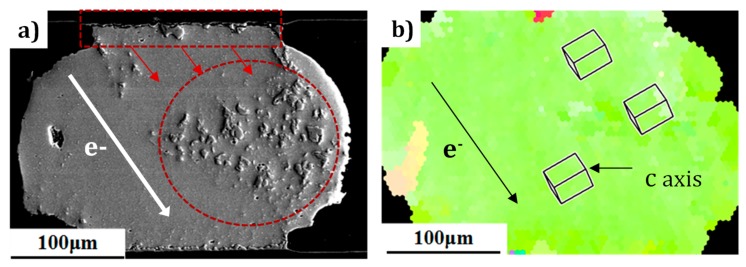
Microstructure images of the microbump with monocrystalline structure stressed at RT. (**a**) SEM images of the Sn3.0Ag0.5Cu microbump after stressing at the current density of 1 × 10^4^ A/cm^2^ for 163.5 h at RT (25 °C) and (**b**) corresponding inverse pole figure of the microbump in Figure 3e.

It has been reported that Cu/Pb/Sn atoms have extremely anisotropic diffusion in Sn grains at RT [33]. We performed a crystallography and micromorphology analysis to correlate the relationship of the Pb atoms migration and the structure of the Sn grains during current stressing. The SEM morphology of the microbump after EM at −196 °C and 25 °C are shown separately in Figure 5 and Figure 6. Significantly, distinct migration patterns were observed in the two bumps. It was found that Pb atoms (identified by EDS), stressed in CT, migrated strangely in a parallel path trajectory, and the paths in different parallel directions formed an angle of approximately 90 degrees. On the contrary, EM at room temperature displayed a rapid Pb migration along a certain directional path, as shown in Figure 6. Figure 5b is the inverse pole figure (IPF), illustrating the crystallographic orientation of the microbump in Figure 5a. The schematic unit cell of a Sn grain represents the orientation of an Sn grain in Figure 5d. It has been reported in previous studies that the metal atoms diffuse much faster along the c-axis than the other two long axes (a-axis or b-axis) in a β-Sn grain lattice [19]. The microbump (β-Sn) has a body-centered tetragonal grain structure at RT. The β-Sn lattice parameters were a = b = 0.583 nm and c = 0.318 nm. This type of lattice has anisotropic properties in electrical conductivity. The resistivity along the a and b axes is 13.25 μΩ-cm, while the c-axis is 20.27 μΩ-cm. [19]. For instance, the calculated anisotropy ratio for Ni could reach as high as 30,000 [34]. Thus, it was not surprising to see that outdiffusion of Cu (or IMC) was accelerated because the c-axis of the grain was almost aligned with the current flow direction in the monocrystalline structure (seen in Figure 6a). Comparatively, since the tin-based solder underwent a phase transition from β-Sn to α-Sn at a temperature lower than 13.2 °C [35], the directionality of the diffusion of Pb elements exhibited significant discrepancy for the two microbumps of single-crystal structure. The analysis of the lattice constants indicated the body-centered tetragonal structure (β-Sn) was anisotropic with respect to the diffusivity, resistivity, and mechanical properties, while the face-centered cubic structure (α-Sn) was isotropic in these properties. Consequently, when the bump was stressed at −196 °C, the grain structure of the microbump was slowly transformed from the β-Sn lattice to the α-Sn lattice, as described in Figure 5d. In the morphological representation (Figure 5a), Pb (identified by EDS) atoms stressed in CT migrated and accumulated in a parallel path trajectory, and the 90 degree angle between the different parallel paths corresponded to the same 90 degree angle between every two lattice axes. For a more detailed analysis of the microstructural variation of the solder joints, enlarged views of the rectangular regions in Figure 5a are shown in Figure 5c.

**Figure 7:**
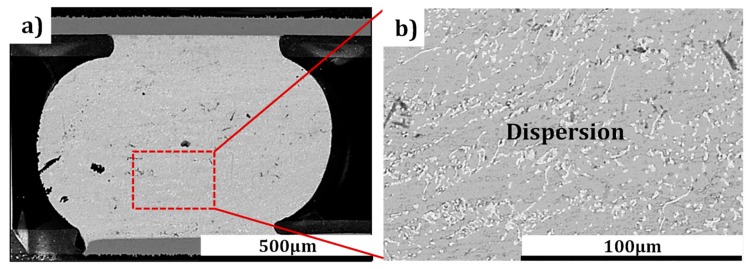
(**a**) and (**b**) are SEM images of the SnPb (Pb content is 22.46%) stored at CT (−196 °C) for 10 days without current stressing.

In this study, another batch of solder bumps were subjected to a cryogenic storage test for 10 days to analyze the driving force of Pb elements’ migration. As can be seen from Figure 7a,b, the Pb atoms (identified by EDS) were uniformly dispersed in the solder matrix, which was completely different from the situation described in Figure 5a,c. This result concluded that it was the current stressing that was the driving force behind Pb migration and accumulation in a regular parallel distribution, rather than the cryogenic temperature.

**Figure 8:**
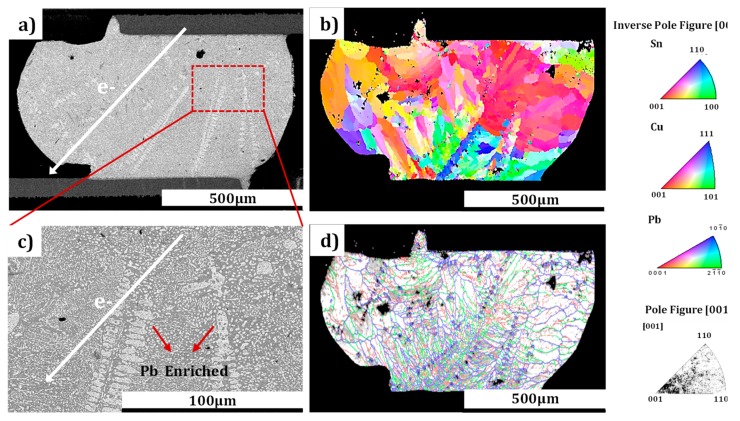
SEM images of Pb migration in a microbump of polycrystalline structure at CT. (**a**) and (**c**) are SEM images of the SnPb microbump (Pb content is 37%) after stressing at the current density of 2.5 × 10^3^ A/cm^2^ for 100 h at CT (−196 °C); (**b**) corresponding inverse pole figure of the microbump in Figure 8a; and (**d**) characterization of the grain boundaries in the microbump of Figure 8a.

In this study, EBSD and SEM characterization analyses of the polycrystalline microbumps were conducted. Compared to the monocrystalline structure, completely different EM behavior occurred in the solder joints of the polycrystalline structure. Long-range migration and accumulation of Pb atoms (identified by EDS) occurred inside the polycrystalline bump at CT, as shown in Figure 8a and the detailed SEM image of Figure 8c. The EBSD orientation images were color coded with the grain orientation, as shown in the accompanying inverse pole figure in Figure 8b, while detailed grain boundaries in this bump is illustrated in Figure 8d. The grain structure of the bump was transformed from β-Sn (tetrahedral structure) to α-Sn (face-centered cubic structure) after stressing at CT. The grain boundaries were high-energy storage regions in this polycrystalline bump, and the diffusion behavior of metal atoms in the face-centered cubic lattice performed isomorphism [36]. As a result, the Pb atoms of the bump gathered at grain boundaries by diffusing through the face-centered cubic lattices around the long grain boundary, and eventually formed the long-range distribution and accumulation of Pb elements shown in Figure 8c. Contrastively, the metal atoms migrated and accumulated in a parallel direction in the bumps of monocrystalline structure. In addition, the grain boundaries in the polycrystalline microbumps were shown to be intertwined, as reflected in the labeled lines of Figure 8d. This structure can, to some extent, suppress the occurrence of EM [37].

## 4. Conclusions

(1) Electromigration was shown to occur at the cathode Cu/solder interface without the effect of Joule heating. Although electromigration does not emerge strikingly at −196 °C compared to that at 25 °C, the rapid growth of (Cu_x_,Ni_1−x_)_6_Sn_5_ IMC was observed at the anomalous region of the cathode interface due to the effect of current crowding.

(2) The isotropic migration behavior of Pb atoms was characterized by the phase transition from the anisotropic β-Sn to the isotropic α-Sn in the lattice structure in the cryogenic environment. A similar diffusion behavior occurs in all directions along α-Sn. This is significantly different from the usual electromigration behavior of diffusion along the c-axis of the β-Sn lattice. 

(3) The grain boundary was a high-energy storage region in polycrystalline bumps, and the diffusion behavior of Pb atoms in face-centered cubic lattices performed isomorphism. As a result, the Pb atoms of the bump gathered at grain boundaries by diffusing through the face-centered cubic lattices around the long grain boundary, eventually forming a long-range distribution and accumulation of Pb elements.

## References

[1] Colin A. (2013). Thermal properties of two materials commonly used in low temperature laboratories. J. Low Temp. Phys..

[2] Macdonald M., Mcinnes C., Hughes G. (2012). Technology requirements of exploration beyond neptune by solar sail propulsion. J. Space Rocket..

[3] Lupinacci A., Shapiro A.A., Suh J.O., Minor A.M. A study of solder alloy ductility for cryogenic applications. Proceedings of the ISAP Conference Proceedings.

[4] Abtew M., Selvaduray G. (2000). Lead-free solders in microelectronics. Mater. Sci. Eng..

[5] Sharma A., Baek B.G., Jung J.P. (2015). Influence of la 2 o 3 nanoparticle additions on microstructure, wetting, and tensile characteristics of sn–ag–cu alloy. Mater. Des..

[6] Nguyen V.L., Kim H.K. (2015). Effect of thermal aging on the mechanical properties of sn3.0ag0.5cu/cu solder joints under high strain rate conditions. J. Electron. Mater..

[7] Andersson C., Sun P., Liu J. (2008). Tensile properties and microstructural characterization of sn–0.7cu–0.4co bulk solder alloy for electronics applications. J. Alloy. Compd..

[8] Tong A.N., Fei Q. (2014). Effects of the intermetallic compound microstructure on the tensile behavior of sn3.0ag0.5cu/cu solder joint under various strain rates. Microelectron. Reliab..

[9] Nguyen V.L., Kim H.K. (2014). Mechanical properties of lead-free solder joints under high-speed shear impact loading. J. Electron. Mater..

[10] Liang S.W., Chen C., Han J.K., Xu L.H., Tu K.N., Lai Y.S. (2010). Blocking hillock and whisker growth by intermetallic compound formation in Sn-0.7Cu flip chip solder joints under electromigration. J. Appl. Phys..

[11] Ren F., Nah J.W., Tu K.N., Xiong B., Xu L., Pang J.H.L. (2006). Electromigration induced ductile-to-brittle transition in lead-free solder joints. Appl. Phys. Lett..

[12] Chen J.Q., Guo J.D., Liu K.L., Shang J.K. (2013). Dependence of electromigration damage on sn grain orientation in sn–ag–cu solder joints. J. Appl. Phys..

[13] Gan H., Tu K.N. (2005). Polarity effect of electromigration on kinetics of intermetallic compound formation in pb-free solder v-groove samples. J. Appl. Phys..

[14] Tian T., Feng X., Han J.K., Choi D., Yin C., Helfen L., Michiel M., Baumbach T., Tu K.N. (2011). Rapid diagnosis of electromigration induced failure time of pb-free flip chip solder joints by high resolution synchrotron radiation laminography. Appl. Phys. Lett..

[15] Nah J.W., Fei R., Tu K.N., Venk S., Camara G. (2006). Electromigration in pb-free flip chip solder joints on flexible substrates. J. Appl. Phys..

[16] Chan Y.C., Yang D. (2010). Failure mechanisms of solder interconnects under current stressing in advanced electronic packages. Prog. Mater. Sci..

[17] Lu M., Shih D.Y., Lauro P., Goldsmith C., Henderson D.W. (2008). Effect of sn grain orientation on electromigration degradation mechanism in high sn-based pb-free solders. Appl. Phys. Lett..

[18] Wang Y., Lu K.H., Gupta V., Stiborek L., Shirley D., Chae S.H., Im J., Ho P.S. (2012). Effects of sn grain structure on the electromigration of sn-ag solder joints. J. Mater. Res..

[19] Chen H., Hang C., Fu X., Li M.Y. (2015). Microstructure and grain orientation evolution in sn-3.0ag-0.5cu solder interconnects under electrical current stressing. J. Electron. Mater..

[20] Tong H.M., Tong H.M. (2013). Advanced Flip Chip Packaging.

[21] Chen H., Han J., Jue L.I., Mingyu L.I. (2012). Inhomogeneous deformation and microstructure evolution of sn-ag-based solder interconnects during thermal cycling and shear testing. Microelectron. Reliab..

[22] Zhou B., Bieler T.R., Lee T.K., Liu K.C. (2010). Crack development in a low-stress pbga package due to continuous recrystallization leading to formation of orientations with [001] parallel to the interface. J. Electron. Mater..

[23] Bieler T.R., Jiang H., Lehman L.P., Kirkpatrick T., Cotts E.J., Nandagopal B. (2008). Influence of sn grain size and orientation on the thermomechanical response and reliability of pb-free solder joints. IEEE Trans. Compon. Packag. Technol..

[24] Zhou B., Quan Z., Bieler T.R., Lee T.K. (2015). Slip, crystal orientation, and damage evolution during thermal cycling in high-strain wafer-level chip-scale packages. J. Electron. Mater..

[25] Zachariasz P., Skwarek A., Illés B., Żukrowski J., Hurtony T., Witek K. (2018). Mössbauer studies of β → α phase transition in sn-rich solder alloys. Microelectron. Reliab..

[26] Maio D.D., Hunt C. (2009). Time-lapse photography of the β-sn/α-sn allotropic transformation. J. Mater. Sci. Mater. Electron..

[27] Plumbridge W.J. (2007). Tin pest issues in lead-free electronic solders. J. Mater. Sci. Mater. Electron..

[28] Leodolter-Dworak M., Steffan I., Plumbridge W.J., Ipser H. (2010). Tin pest in sn-0.5cu lead-free solder alloys: A chemical analysis of trace elements. J. Electron. Mater..

[29] Skwarek A., Illés B., Horváth B., Géczy A., Zachariasz P., Bušek D. (2017). Identification and characterization of β→α-sn transition in sncu1 bulk alloy inoculated with insb. J. Mater. Sci. Electron..

[30] Yeh C.C., Choi W.J., Tu K.N., Elenius P., Balkan H. (2002). Current-crowding-induced electromigration failure in flip chip solder joints. Appl. Phys. Lett..

[31] Huntington H.B., Grone A.R. (1961). Current-induced marker motion in gold wires. J. Phys. Chem. Solids.

[32] Wu A.T., Tu K.N., Lloyd J.R., Tamura N., Valek B.C., Kao C.R. (2004). Electromigration-induced microstructure evolution in tin studied by synchrotron x-ray microdiffraction. Appl. Phys. Lett..

[33] Liu P., Wang S., Li D., Li Y., Chen X.Q. (2016). Fast and huge anisotropic diffusion of cu (ag) and its resistance on the sn self-diffusivity in solid beta-sn. J. Mater. Sci. Technol..

[34] Yang T.L., Yu J.J., Li C.C., Lin Y.F., Kao C.R. (2015). Dominant effects of sn orientation on serrated cathode dissolution and resulting failure in actual solder joints under electromigration. J. Alloy Compd..

[35] Chiavari C., Martini C., Poli G., Prandstraller D. (2006). Deterioration of tin-rich organ pipes. J. Mater. Sci..

[36] Tasooji A., Lara L., Lee K. (2014). Effect of grain boundary misorientation on electromigration in lead-free solder joints. J. Electron. Mater..

[37] Genut M., Li Z., Bauer C.L., Mahajan S., Tang P.F., Milnes A.G. (1991). Characterization of the early stages of electromigration at grain boundary triple junctions. Appl. Phys. Lett..

